# Integrated analysis of potential microbial consortia, soil nutritional status, and agro-climatic datasets to modulate P nutrient uptake and yield effectiveness of wheat under climate change resilience

**DOI:** 10.3389/fpls.2022.1074383

**Published:** 2023-01-12

**Authors:** Mahreen Yahya, Maria Rasul, Sayed Zajif Hussain, Adil Dilawar, Midrar Ullah, Lubna Rajput, Aftab Afzal, Muhammad Asif, Tesfaye Wubet, Sumera Yasmin

**Affiliations:** ^1^Soil and Environmental Biotechnology Division, National Institute for Biotechnology and Genetic Engineering College, Pakistan Institute of Engineering and Applied Sciences (NIBGE-C, PIEAS), Punjab, Pakistan; ^2^Department of Environment and Energy, Sejong University, Neungdong-ro, Gwangjin-gu, Republic of Korea; ^3^Department of Chemistry and Chemical Engineering, Syed Babar Ali-School of Science and Engineering (SBA-SSE), Lahore University of Management Sciences (LUMS), Punjab, Pakistan; ^4^State Key Laboratory of Resources and Environmental Information System, Institute of Geographic Sciences and Natural Resources Research, Chinese Academy of Sciences, Beijing, China; ^5^University of Chinese Academy of Sciences (UCAS), Beijing, China; ^6^Department of Biotechnology, Shaheed Benazir Bhutto University, Khyber Pakhtunkhwa, Pakistan; ^7^Plant Physiology and Biotechnology Agricultural Research Centre, Sindh, Pakistan; ^8^Department of Botany, Hazara University Mansehra, Khyber Pakhtunkhwa, Pakistan; ^9^Agricultural Biotechnology Division, National Institute for Biotechnology and Genetic Engineering College, Pakistan Institute of Engineering and Applied Sciences (NIBGE-C, PIEAS), Punjab, Pakistan; ^10^Department of Community Ecology, Helmholtz Centre for Environmental Research (UFZ), Halle, Germany; ^11^German Centre for Integrative Biodiversity Research (iDiv) Halle-Jena-Leipzig, Leipzig, Germany

**Keywords:** soil-specific consortia, rhizoscanning, soil organic matter, root architecture, climatic conditions, fluorescence *in situ* hybridization, field emission scanning electron microscopy

## Abstract

Climate change has a devastating effect on wheat production; therefore, crop production might decline by 2030. Phosphorus (P) nutrient deficiency is another main limiting factor of reduced yield. Hence, there is a dire need to judiciously consider wheat yield, so that human requirements and nutrition balance can be sustained efficiently. Despite the great significance of biostimulants in sustainable agriculture, there is still a lack of integrated technology encompassing the successful competitiveness of inoculated phosphate-solubilizing bacteria (PSB) in agricultural systems in the context of climatic conditions/meteorological factors and soil nutritional status. Therefore, the present study reveals the modulation of an integrated P nutrient management approach to develop potential PSB consortia for recommended wheat varieties by considering the respective soil health and agro-climatic conditions. The designed consortia were found to maintain adequate viability for up to 9 months, verified through field emission scanning electron microscopy and viable count. Furthermore, a significant increase in grain yield (5%–8%) and seed P (4%) content was observed in consortia-inoculated wheat plants with 20% reduced Diammonium phosphate (DAP) application under net house conditions. Fluorescence *in situ* hybridization analysis of roots and amplification of the *gcd* gene of *Ochrobactrum* sp. SSR indicated the survival and rhizosphere competency of the inoculated PSB. Categorical principal component analysis (CAT-PCA) showed a positive correlation of inoculated field-grown wheat varieties in native soils to grain yield, soil P content, and precipitation for sites belonging to irrigated plains and seed P content, soil organic matter, and number of tillers for sites belonging to Northern dry mountains. However, the impact of inoculation at sites belonging to the Indus delta was found significantly correlated to soil potassium (K) content, electrical conductivity (EC), and temperature. Additionally, a significant increase in grain yield (15%) and seed P (14%) content was observed in inoculated wheat plants. Thus, the present study demonstrates for the first time the need to integrate soil biological health and agro-climatic conditions for consistent performance of augmented PSB and enhanced P nutrient uptake to curtail soil pollution caused by the extensive use of agrochemicals. This study provides innovative insights and identifies key questions for future research on PSB to promote its successful implementation in agriculture.

## Introduction

Global warming is causing a rapid increase in the Earth’s surface temperature, leading agriculture to face multiple challenges (Camaille et al., 2021). In addition, climate change is the major uncontrollable factor, adversely affecting global food production (Farooq et al., 2022). Climate change is likely to increase the intensity of extreme climate events that will affect patterns of agricultural production, water cycle, and eventually food security (Muluneh, 2021). All of these events resulted in increasing floods, declining food production and quality, and increasing food prices. Such events caused substantial yield losses in major cereal crops such as 5.5% yield reduction in wheat (Shah et al., 2021). On the other hand, global food demand is expected to increase by 60% with the increasing global population (Bijl et al., 2018). Therefore, there is a pressing need at this crucial time to transit toward sustainable crop production that enables crops to grow well under resource-limited environmental challenging conditions with optimum yields across a wide array of environmental conditions (Reynolds et al., 2021).

Today, novel and advanced techniques such as smart irrigation, fertilizers with enhanced efficiency, integration fertilizers, and pest management have been adapted for sustainable crop production (Ahmad et al., 2022). The challenge faced by 40% of the global phosphorus (P)-deficient soil has been addressed by the application of phosphatic chemical fertilizers in recent years. However, most of these chemical fertilizers applied to the soil become unavailable to the plant, and their excessive application to overcome the P deficiency leads to environmental pollution concerning contamination of groundwater and eutrophication (Alori et al., 2017). Rock phosphate (RP), on the other hand, is the primary source of P, but it is a nonrenewable resource that is progressively exhausted worldwide (Pavinato et al., 2022). RP has low agronomic effectiveness due to its crude nature, high reactivity, and less solubility in the soil (Soumare et al., 2020).

Integration of plant growth-promoting bacteria in agriculture biotechnology represents a promising solution for improved soil fertility and crop yield. A group of microorganisms called phosphate-solubilizing bacteria (PSB) is the key component to increasing the availability of insoluble P for plant use. Nowadays, PSB-based biofertilizers are considered crucial constituents that contribute to sustainable production in agro-ecosystems (Mitter et al., 2021). The persistence of PSB is the most important underlying factor in designing successful bioinoculants because it indicates the interaction of the microbial inoculation with the host plant, its capability to compete with indigenous microbes and cope with abiotic conditions that depend on soil type, its characterization, and agro-climatic conditions (Finkel et al., 2017). Therefore, designing climate-smart biofertilizers and evaluating their persistence in native soil and climate would be a potential approach to boost plant growth and substantial resilience in agriculture.

To the best of our knowledge, a holistic approach to disentangle the system in the context of climatic conditions/meteorological factors and soil nutritional status is scarce. As previous microbial inocula are either being evaluated under controlled conditions (Chen and Liu, 2019; Elhaissoufi et al., 2020) or if conducted under field conditions (Sedri et al., 2022; Liu et al., 2022), no emphasis is usually given to the native microbial bacteria, soil nutrient status, and climatic conditions of the agricultural site. Most of the previous studies are based on identifying microbes in the wheat rhizosphere and application of the same bacteria to different environments (Mahoney et al., 2017; Ullah et al., 2022; Sedri et al., 2022); however, studies on the development of soil-specific PSB consortia and their application in respective climatic zones are entirely missing.

Hence, the present study is the first comprehensive report in which soil-specific consortia were developed, composed of indigenous PSB from wheat-growing agro-climatic zones of Pakistan, and implemented in their respective soils under field conditions for wheat. It was thus hypothesized (H1) that the application of soil-specific consortium along with recommended wheat varieties can improve wheat yield predominantly by relating to the soil nutritional status and meteorological conditions to ensure the survival of inoculated native PSB in the wheat rhizosphere. A positive correlation might exist (H2) between meteorological and soil nutritional factors and wheat yield that might result in improved wheat production.

## Materials and methods

### Sample collection, soil physicochemical analysis, and environmental data

Soil samples were collected from different provinces of Pakistan. Province 1, i.e., Site 1: Faisalabad (31°23’45.1”N, 73°01’3.4”E), Site 2: Nankana Sahib (31°27′0″N, 73°42′24″E), and Site 3: Pindi Bhattian (31°6’954”N, 73°18’66”E); Province 2, i.e., Site 4: Hazara (34°25′12″N, 73°15′0″E); and Province 3, i.e., Site 5: Husri (25°19′0″N, 68°25′0″E) and Site 6: Tando Jam (25°25′40.21″N, 68°31′40.4″E) were selected for the study. Soil samples from each site were collected at the depth of 20 cm and analyzed for soil physicochemical properties. Soil pH and electrical conductivity (EC) were measured using a pH meter (PHS-3C, REX, Shanghai) and an electrical conductivity meter (DDS-307A, REX, Shanghai), respectively (Rhoades, 1993). The wet oxidation method was used to determine soil organic matter (Nelson and Sommers, 1996). Soil total nitrogen (N) was determined by the Kjeldahl method (Bremner and Mulvaney, 1983). Soil-available P was measured by the sodium bicarbonate method (Olsen, 1954). Sodium content and soil exchangeable potassium (K) were determined using a flame photometer (Model 410, Corning, Halstead, UK; Simard, 1993). Meteorological data of each site were collected from Pakistan Meteorological Department (PMD) (https://www.pmd.gov.pk/en/).

### Bacterial strains used

Bacterial strains used in this study are a subset of a large collection of PSB isolated from the rhizosphere soil of wheat grown in different agro-ecological zones of Pakistan (Yahya et al., 2021). PSB, i.e., *Bacillus* sp. TAYB, *Enterobacter* spp. ZW9, *Enterobacter* spp. ZW32, *Enterobacter* spp. D1, *Ochrobactrum* sp. SSR, *Pantoea* sp. S1, and *Pseudomonas* sp. TJA were used in the study for consortium development and obtained from the National Institute for Biotechnology and Genetic Engineering (NIBGE) Biotech Resource Center (NBRC: http://www.nibge.org/Default.aspx). The 16S rRNA gene sequences of these strains were deposited to NCBI GenBank (https://www.ncbi.nlm.nih.gov/). *Enterobacter* spp. ZW32 (accession number: MK817561), *Ochrobactrum* sp. SSR (accession number: MK422612), and *Enterobacter* spp. ZW9 (accession number: MK024209) were isolated from Province 1 (Punjab). *Enterobacter* spp. D1 (accession number: MK422618) and *Pantoea* sp. S1 (accession number: MK422619) were isolated from Province 2 [Khyber Pakhtunkhwa (KPK)]. While *Bacillus* sp. TAYB (accession number: MN754081) and *Pseudomonas* sp. TJA (accession number: MK422620) were isolated from Province 3 (Sindh).

All of the strains used in the present study have multiple plant growth-promoting attributes, i.e., phosphate solubilization, zinc solubilization, indole acetic acid production, and organic acid production (Yahya et al., 2021).

### Development of bioformulation with soil-specific consortia

Three different consortia were designed by selecting soil-/site-specific PSB for recommended wheat varieties to that particular site. Wheat variety-1 Faislabad-2008 recommended for Province 1 (Punjab) was inoculated with consortium-1, i.e., *Enterobacter* spp. ZW32, *Ochrobactrum* sp. SSR, and *Enterobacter* spp. ZW9. Consortium-2 comprising *Enterobacter* spp. D1, *Ochrobactrum* sp. SSR, and *Pantoea* sp. S1 was designed for wheat variety-2 (Fakhr-e-Sarhad) recommended for Province 2 (KPK). Whereas consortium-3 comprising *Bacillus* sp. TAYB, *Ochrobactrum* sp. SSR, and *Pseudomonas* sp. TJA was used for wheat variety-3 (TD1) recommended for Province 3 (Sindh).

For the preparation of the inoculum, a loopful of each bacterial culture was transferred to 25 ml of Luria-Bertani (LB) broth medium separately and grown anaerobically on a rotatory shaker at 28°C ± 2°C for 24 to 48 h. Bacterial cultures for each consortium were mixed separately to make a suspension (1 × 10^9^ CFU ml^-1^).

Three filter mud (FM) and soil-specific consortia-based bioformulations were developed. FM, an agro-industrial by-product of sugar cane (Yahya et al., 2022), was ground and sieved through a 2-mm sieve and further autoclaved before inoculation. The bacterial suspension (300 ml) of each consortium (1 × 10^9^ CFU ml^-1^) was then aseptically and uniformly mixed in 700 g of FM, packed in polythene bags, and incubated at 28°C (Pastor-Bueis et al., 2019). Uninoculated control was prepared by mixing LB broth (300 ml) with sterilized FM (700 g).

### Field emission scanning electron microscopy of bioformulations

Field emission scanning electron microscopy (FESEM) was used to assess the presence of inoculated PSB in the tested formulations up to 270 days post-inoculation (DPI). Furthermore, the viability of inoculated bacteria was estimated from each bioformulation on LB agar medium and National Botanical Research Institute’s phosphate (NBRIP) agar medium by the serial dilution method (Nautiyal, 1999).

### Evaluation of soil-specific consortia in earthen pots under net house conditions

The effect of three bioformulations was assessed on wheat variety Faisalabad 2008 using native soil collected from Faisalabad (loamy soil texture, available P 1.87 mg kg^-1^, organic matter 0.57%, and pH 8) in earthen pots under net house conditions. Seeds were sterilized with 1.5% sodium hypochlorite (NaOCl) solution for 5 min and washed with autoclaved sterilized water five times. Sterilized seeds were pelleted with bioformulation (2 kg of carrier per 50 kg seeds) comprising respective consortium suspension (1 × 10^9^ CFU ml^-1^). Seeds were then incubated for 30 min. Seeds pelleted with uninoculated sterilized FM were used as controls. Six seeds were sown per pot (30 cm diameter) containing 5 kg of soil and arranged in a completely randomized design. All inoculated treatments were supplemented with 80% DAP, i.e., 20% reduced amount of DAP, and two uninoculated controls supplemented with 80% or 100% DAP.

### Measurement of plant growth parameters and soil nutrient analysis

Plants were uprooted after 35 DPI to evaluate plant growth parameters, i.e., root length, shoot length, and dry weight of plant. Plants were harvested at maturity, and data regarding plant height, number of tillers, grain yield, plant biomass, and plant P content (Tandon, 1993) were recorded. Six plants were selected from each replicate of each treatment for analysis. Rhizospheric soil was analyzed for available P by the molybdenum blue method (Olsen, 1954) and alkaline phosphatase activity by p-nitrophenyl method (Tabatabai and Bremner, 1969).

#### Detection of inoculated Phosphate Solubilizing bacteria (PSB)

The survival of inoculated Phosphate Solubilizing bacteria (PSB) was assessed by viable count (Somasegaran and Hoben, 2012). Root colonization and persistence of inoculated PSB were studied by fluorescence *in situ* hybridization (FISH). FLUOS-labeled green probe EUB338 was used to detect the PSB population. Reisolated colonies of PSB were identified by comparing morphological characteristics and P solubilization to that of pure colonies (Yasmin et al., 2016). Morphologically similar colonies of SSR obtained from all inoculated treatments were further validated by amplification of the *gcd* gene (MK883703) specific for *Ochrobactrum* strain SSR (Rasul et al., 2021).

#### Evaluation of soil-specific consortia for wheat yield parameters in multilocational field trials

**Figure 1:**
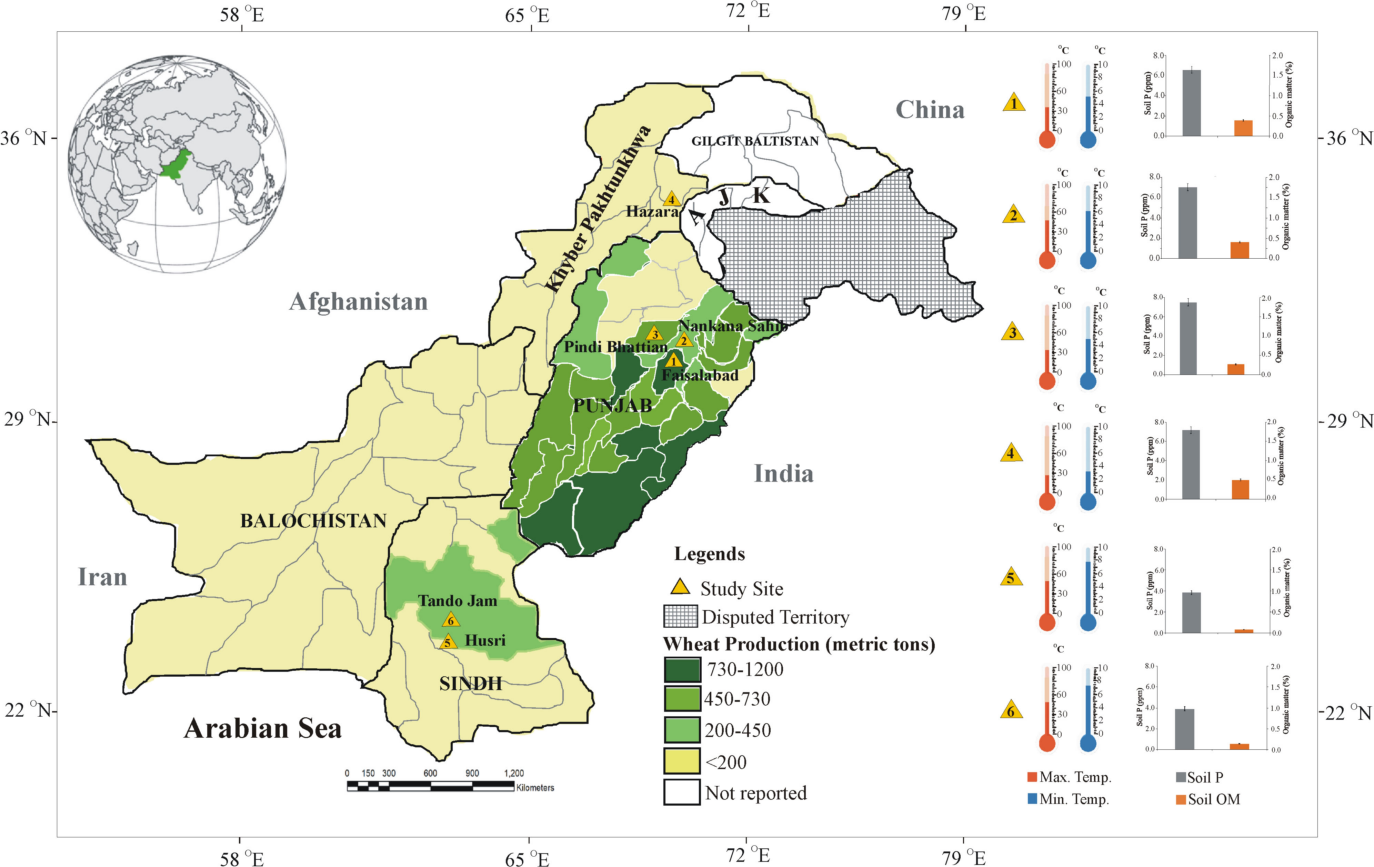
Map of Pakistan with multilocation trials targeted in the present study for evaluation of soil-specific consortia on recommended wheat varieties. Different shades of green colors highlight the region showing wheat production in the main wheat districts of Pakistan (source: https://ipad.fas.usda.gov/countrysummary/Default.aspx?id=PK&crop=Wheat). Thermometers and bar graphs show climatic temperature (Pakistan Meteorological Department; source: https://www.pmd.gov.pk/en/) soil available P and organic matter of the soil of study sites.

The developed consortia were further evaluated under different wheat-growing agro-climatic field conditions during the winter season of 2019–2020 in Province 1 (Punjab), i.e., NIBGE field, Faisalabad, Pindi Bhattian, Nankana Sahib; Province 2 (KPK), i.e., Hazara; and Province 3 (Sindh), i.e., Husri and Tando Jam (**Figure 1**). All three consortia were prepared as described in the above section. Bacterial cultures for each consortium were mixed separately to make a suspension (1 × 10^9^ CFU ml^-1^), then mixed uniformly with FM (700 g) and incubated at 28°C (Pastor-Bueis et al., 2019). Three treatments composed of consortium-inoculated seeds supplemented with 80% of the recommended dose of DAP (i.e., 20% reduced DAP) and two uninoculated controls supplemented with 80% or 100% of DAP (i.e., recommended dose of DAP) were considered for multilocation trials.

Experiments were carried out in a randomized complete block design. Each treatment consisted of three replicates and a plot size of 4 m × 6 m at NIBGE, 4 m × 4 m at Pindi Bhattian, 6 m × 6 m at Nankana Sahib and Hazara, and 4 m × 5 m at Husri and Tando Jam. Seeds were sown by the drill method using a hand drill. The experiment was conducted under standard agronomic practices. At maturity, plants were harvested and data were recorded for grain yield, plant biomass, harvest index (HI), plant height, number of tillers, and seed P. Soil-available P (Olsen, 1954) and alkaline phosphatase activity (Tabatabai and Bremner, 1969) were determined according to standard protocols.

### Statistical analysis

Data were statistically analyzed using ANOVA. Least significant difference (LSD) compared variations between the treatments at a 5% level of confidence using Statistix 10 software (Tallahassee, FL, USA). Principal component analysis (PCA) was performed using SPSS 23.0 software (SPSS Inc., USA).

## Results

### Soil physicochemical analysis and environmental data

Biochemical analysis of soil parameters revealed the difference in soil properties for all of the soils from five different sites belonging to major wheat-growing areas (**Table 1**). Organic matter of the soils from the Indus delta (Sindh) was below 1%. While in soils from the Northern irrigated plains (Punjab) and in particular from the Northern dry mountains Khyber Pakhtunkhwa (KPK), the organic matter was up to 1.7%. As Pakistani soils are alkaline calcareous in nature, the pH of the soils ranged from 7 to 8.5. Soil EC was much higher in soils belonging to the Indus delta (3.42 dS m^-1^) and lower in soils of the Northern dry mountains (1.1 dS m^-1^). Whereas soil N, P, and K content were higher in the KPK soils and much lower in soils belonging to Sindh.

**Table 1:** Physicochemical properties of soils collected and environmental data of the experimental field sites.

	Parameters	Experimental sites					
		Site 1	Site 2	Site 3	Site 4	Site 5	Site 6
		Faisalabad	Nankana Sahib	Pindi Bhattian	Hazara	Husri	Tando Jam
**Physico-chemical Properties**	pH	7.94 ± 0.41	7.98 ± 0.42	7.62 ± 0.38	7.50 ± 0.52	8.20 ± 0.41	8.50 ± 0.43
	EC (dS m^-1^)	1.25 ± 0.06	1.51 ± 0.08	1.45 ± 0.07	1.10 ± 0.05	2.16 ± 0.41	3.42 ± 0.17
	Organic matter (%)	1.57 ± 0.03	1.60 ± 0.03	1.06 ± 0.03	1.71 ± 0.05	0.32 ± 0.04	0.56 ± 0.05
	Available P (µg kg^-1^)	4.21 ± 0.29	4.73 ± 0.36	4.45 ± 0.31	4.60 ± 0.31	3.27 ± 0.22	3.52 ± 0.28
	Total N (%)	0.030 ± 0.002	0.327 ± 0.002	0.031 ± 0.001	0.042 ± 0.003	0.017 ± 0.001	0.025 ± 0.001
	Extractable K (mg kg^-1^)	153 ± 7.65	149 ± 7.45	129 ± 6.45	125 ± 6.57	181 ± 9.05	145 ± 7.25
	Soil Texture	Sandy Loam	Loam	Sandy Loam	Loam	Clay Loam	Clay Loam
**Climatic Conditions**	Rain fall (mm)	225.67 ± 11.28	254.50 ± 12.73	244.50 ± 12.23	166.43 ± 8.32	7.53 ± 0.38	8.07 ± 0.40
	Average min Temp(°C)	12.08 ± 0.60	11.75 ± 0.59	11.49 ± 0.57	9.80 ± 0.49	13.23 ± 0.66	13.87 ± 0.69
	Average max Temp(°C)	24 ± 1.20	23.50 ± 1.18	23.50 ± 1.18	23.60 ± 1.18	27.60 ± 1.38	27.73 ± 1.39
	Relative Humidity (%)	62.87 ± 3.14	64.40 ± 3.22	64.17 ± 3.21	63.00 ± 3.15	55.83 ± 2.79	56.17 ± 11.95
	Sunshine Duration (hours/month)	195.33 ± 9.77	183.67 ± 9.18	182.53 ± 9.13	157.37 ± 7.87	239.00 ± 11.95	237 ± 11.85

Physicochemical properties of soils collected from different sites of wheat-growing areas, Pakistan. Values are an average of six biological replicates EC, Eclectic conductivity; KPK, Khyber Pakhtunkhwa.

### Shelf life of the bioformulations

**Figure 2:**
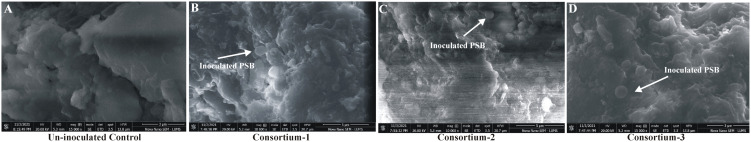
Shelf life study of filter mud-based formulation inoculated with soil-specific consortia under controlled conditions. Field emission scanning electron microscopic (FESEM) analysis of uninoculated filter mud-based bioformulations **(A)** and inoculated filter mud-based bioformulations with consortium-1 **(B)**, consortium-2 **(C)**, and consortium-3 **(D)**.

Three FM-based soil-specific consortia were evaluated for shelf life under controlled conditions. The survival of all PSB included in the three consortia was confirmed up to 270 DPI as indicated by both viable count and visualization of PSB by FESEM (**Figure 2**; **Table S1**). Maximum viability of soil-specific consortia was maintained (up to 2 × 10^9^ CFU ml^-1^) at 90 DPI for consortium-1 (**Table S1**).

### Evaluation of soil-specific consortia in earthen pots under net house conditions

**Figure 3:**
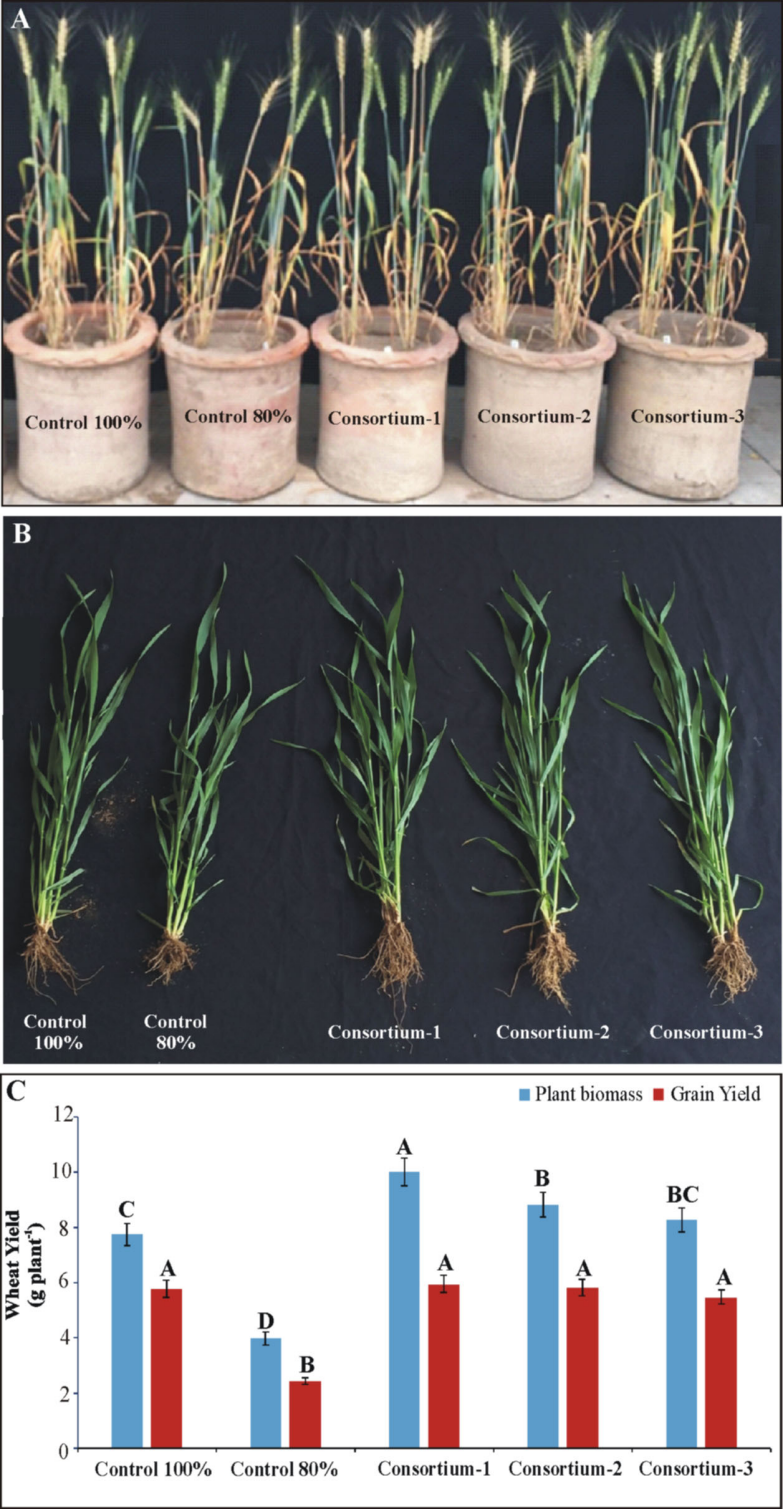
Evaluation of soil-specific consortia for plant yield parameters of wheat grown in pots under net house conditions **(A, B)**. Effects of bioformulation on plant biomass **(C)** and grain yield were recorded. Mean values denoted by the same letter are not significantly different at P = 0.05 according to LSD.

All PSB consortia improved wheat growth significantly in the pot experiment under net house conditions (**Figure 3**). Consortium-1 showed the maximum increase in grain yield (5.95 g plant^-1^) followed by consortium-2 (5.83 g plant^-1^) and consortium-3 (5.77 g plant^-1^) as compared to 80% and 100% uninoculated controls. A significant increase (3.6%–4.1%) in seed P was observed in inoculated plants compared to uninoculated controls. Upon inoculation with the consortia, an increase in available soil P (5.7–6.25 μg g^-1^ soil) and phosphatase activity (22–24 μmol g^-1^ soil h^-1^) was observed (**Table S2**).

**Figure 4:**
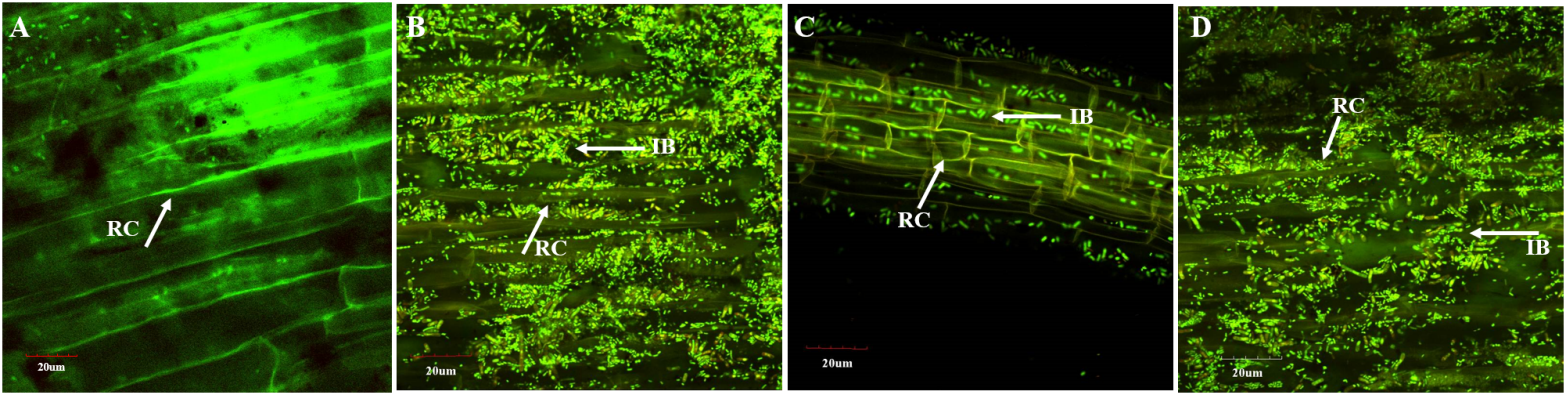
Confocal laser scanning microscopy of wheat roots at 35 days after inoculation of consortia in pot experiment under net house conditions. Oligonucleotide probes labeled with FLUOS dye showed green fluorescent signals for the entire bacterial population in the uninoculated control **(A)** and in wheat inoculated with consortium-1 **(B)**, consortium-2 **(C)**, and consortium-3 **(D)**. IB, inoculated bacteria; RC, root cells.

The presence of inoculated PSB was detected on wheat roots from the earthen pot experiment at 35 DPI (**Figure 4**). The highest density of PSB was observed in roots inoculated with consortium-1 and consortium-3 (**Figures 4B, D**). Reisolated PSB colonies were identified based on their morphological characteristics and phosphate solubilization (233–359 µg ml^-1^). Furthermore, one of the PSB strains, *Ochrobactrum* SSR, was validated by the amplification of the strain-specific *gcd* gene that confirmed the presence of inoculated bacteria in consortia-inoculated soil (**Figure S1**).

### Evaluation of soil-specific consortia on wheat yield parameters in multilocational field trials

Soil-specific consortia improved various plant growth parameters of wheat under respective soil conditions. Maximum grain yield (5,390 kg ha^-1^) was observed as a result of consortium-1 inoculation at site 2 followed by site 3 (5,240 kg ha^-1^) and site 1 (4,806 kg ha^-1^). In the case of consortium-2, grain yield of 5,174 kg ha^-1^ was observed at site 4 with a 20% reduced application of DAP. Maximum grain yield (5,324 kg ha^-1^) was observed as a result of consortium-2 inoculation at site 6 followed by site 5 (4,806 kg ha^-1^). HI ranged from 30% to 36%.

Inoculation of consortium-1 increased (up to 15%) grain yield at site 2 followed by site 3 (12%) and site 1 (2%). However, inoculation of consortium-2 increased the grain yield by 8% at site 4 as compared to 80% control. The increase in grain yield was 5% at site 5 and 14% at site 6 in inoculated treatments with consortium-3 as compared to 80% control (**Table 2**).

**Table 2:** Effect of PSB consortia on various wheat yield and soil parameters in multilocational field trials.

Province	Sites	Districts	Treatments	No. of tillers(tillers m^-2^)	Plant height(cm)	Plant biomass (kg ha^-1^)	Grain yield (kg ha^-1^)	^1^Seed P (%)	^2^Soil Available P	^3^Phosphatase Activity	Harvest Index(%)
**Province 1** **(Punjab)**	**1**	Faisalabad	Inoculated	373 ± 19 A	107 ± 5.51 A	14,500 ± 725 A	4,806 ± 240 A	4.50 ± 0.23 A	6.30 ± 0.31 A	26.33 ± 1.32 A	33
			80% Control	321 ± 16 A	104 ± 5.22 A	14,300 ± 715 A	4,728 ± 236 A	4.00 ± 0.20 B	5.60 ± 0.28 B	23.20 ± 1.16 B	33
			100% Control	338 ± 17 A	105 ± 5.25 A	14,400 ± 720 A	4,789 ± 239 A	4.17 ± 0.21AB	5.97 ± 0.29 AB	24.20 ± 1.21 AB	33
	**2**	Nankana Sahib	Inoculated	480 ± 24 A	110 ± 5.57 A	17,050 ± 852 A	5,390 ± 270 A	4.50 ± 0.23 A	6.37 ± 0.32 A	27.00 ± 1.32 A	32
			80% Control	370 ± 19 B	105 ± 5.00 B	14,333 ± 717 B	4,610 ± 231 B	4.05 ± 0.20 B	5.64 ± 0.28 A	24.20 ± 0.50 B	32
			100% Control	407 ± 20 B	108 ± 5.03 AB	15,250 ± 763 B	4,810 ± 247 AB	4.13 ± 0.21 AB	6.07 ± 0.30 A	25.20 ± 0.96 B	32
	**3**	Pindi Bhattian	Inoculated	340 ± 17 A	109 ± 5.43 A	17,167 ± 858 A	5,240 ± 262 A	4.00 ± 0.20 A	5.33 ± 0.27 A	27.00 ± 1.35 A	31
			80% Control	262 ± 13 B	105 ± 5.25 A	15,167 ± 758 B	4,590 ± 230 B	3.65 ± 0.18 B	4.99 ± 0.25 B	23.63 ± 1.18 B	30
			100% Control	277 ± 14 B	108 ± 5.38 A	16,033 ± 767 B	5,020 ± 251 A	3.81 ± 0.19 AB	5.08 ± 0.25 AB	25.30 ± 1.27 AB	31
**Province 2(KPK)**	**4**	Hazara	Inoculated	440 ± 22 A	107 ± 5.35 A	14,489 ± 724 A	5,174 ± 259 A	3.55 ± 0.18 A	4.52 ± 0.23 A	24.67 ± 1.24 A	36
			80% Control	342 ± 17 B	105 ± 5.25 A	13,051 ± 653 C	4,747 ± 237 C	3.20 ± 0.16 B	4.13 ± 0.21 B	21.33 ± 1.07 B	36
			100% Control	357 ± 18 B	106 ± 5.30 A	13,905 ± 698 B	4,954 ± 248 B	3.25 ± 0.16 B	4.30 ± 0.22 AB	22.00 ± 1.10 AB	36
**Province 3** **(Sindh)**	**5**	Husri	Inoculated	351 ± 19 A	85 ± 4.25 A	15,075 ± 841 A	4,806 ± 240 A	2.91 ± 0.15 A	3.95 ± 0.20 A	20.07 ± 1.00 A	32
			80% Control	322 ± 16 A	82 ± 4.24 A	14,789 ± 703 A	4,550 ± 228 B	2.62 ± 0.13 B	3.58 ± 0.18 B	18.17 ± 0.91 B	31
			100% Control	338 ± 17 A	85 ± 4.12 A	14,855 ± 708 A	4,642 ± 232 B	2.79 ± 0.14 AB	3.61 ± 0.18 B	15.33 ± 0.96 AB	31
	**6**	Tando Jam	Inoculated	373 ± 19 A	89 ± 4.47 A	16,276 ± 813 A	5,324 ± 266 A	3.05 ± 0.15 A	4.50 ± 0.23 A	21.33 ± 1.07 A	33
			80% Control	328 ± 16 B	81 ± 3.75 A	14,089 ± 704 B	4,587 ± 229 B	2.72 ± 0.14 B	4.14 ± 0.21 B	19.67 ± 0.98 B	33
			100% Control	347 ± 17 AB	85 ± 3.77 A	14,389 ± 719 B	4,712 ± 236 B	2.80 ± 0.14 B	4.37 ± 0.22 AB	20.17 ± 1.01 B	33

Data are an average of three replicates.

^1^Plant P content is given in % of total plant weight.

^2^Soil-available P is presented in μg g^-1^ soil.

^3^Soil phosphatase activity is presented in µmoles g^-1^ soil h^-1^.

± represents standard deviation. Means with significant differences (P < 0.05) among treatments are represented by different letters. KPK, Khyber Pakhtunkhwa.

#### Effect of PSB inoculation on plant P content

A significant increase in plant P content was observed as a result of PSB inoculation. The PSB-inoculated treatments showed a significant increase in plant seed P content compared to the 80% and 100% controls. The plant P content was significantly higher (4%–4.9%) in inoculated treatments at sites 1, 2, and 3 followed by sites 4, 6, and 5 (**Table 2**). Maximum plant P content (4.9%) was observed at site 2 followed by site 1 (4.5%) in PSB-inoculated treatment as compared to both 80% and 100% controls, whereas plant P content at site 6 (3.7%) and site 5 (3.5%) also increased as compared to both controls.

#### Effect of PSB inoculation on soil-available P and phosphatase activity

Soil phosphatase activity was also significantly higher in PSB-inoculated treatments. There was a pronounced increase in soil phosphatase activity at sites 2, 3, and 1, followed by sites 4, 6, and 5. Maximum soil phosphatase activity (26 µmoles g^-1^ soil h^-1^) was observed for site 1 in consortium-1-inoculated treatment as compared to both 80% and 100% controls. The maximum soil phosphatase activity (24 µmoles g^-1^ soil h^-1^) was for site 4 in consortium-2-inoculated treatment. While soil phosphatase activity (17 µmoles g^-1^ soil h^-1^) for sites 5 and 6 in consortium-3-inoculated treatment was higher as compared to both 80% and 100% controls (**Table 2**).

### Trends and variations of meteorological factors at multilocational field sites

**Figure 5:**
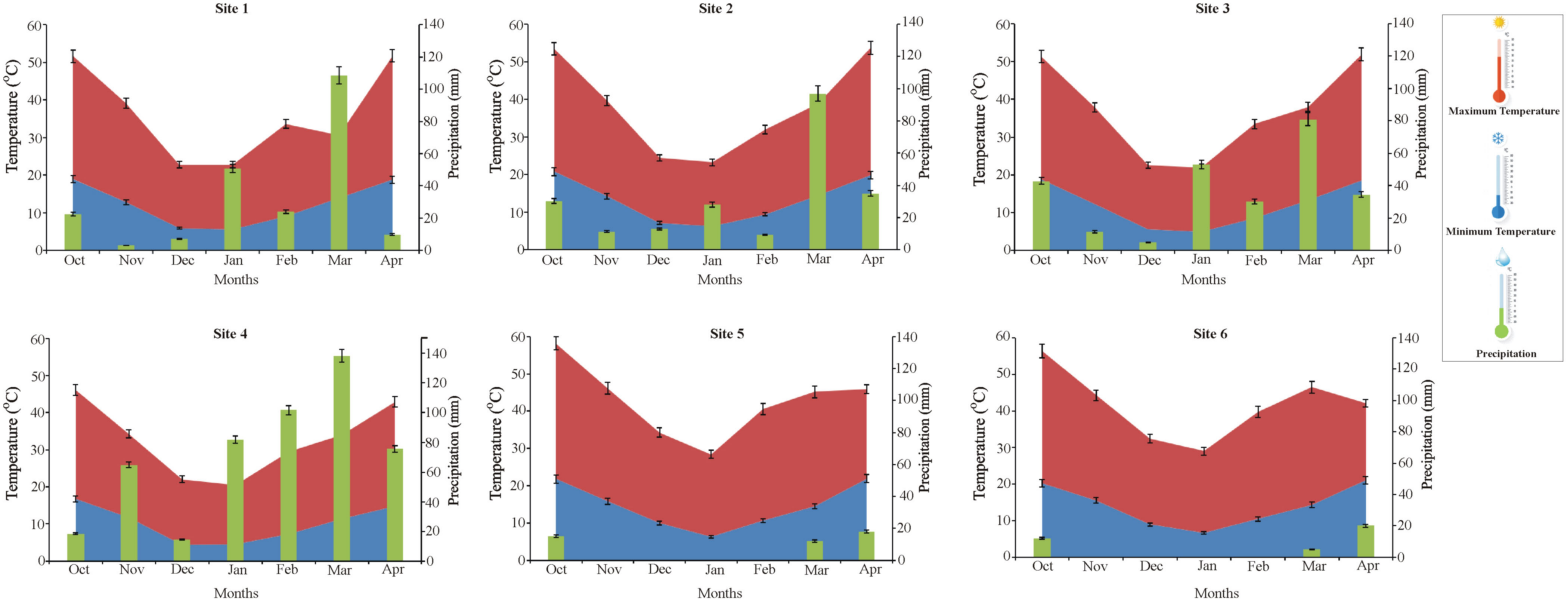
Trends of meteorological factors at multilocation field sites with respect to precipitation (green), minimum temperature (blue) and maximum temperature (red) during the wheat season 2019-20. Site1: Faisalabad, Site 2: Nankana Sahib, Site 3: Pindi Bhattian, Site 4: Hazara, Site 5: Husri and Site 6: TandoJam.

The trends in the change of key meteorological factors were analyzed during the wheat season 2019–2020 at the six field sites with respect to precipitation and temperature (**Figure 4**). Maximum precipitation was recorded at site 4 during the month of March. However, precipitation was recorded during the months of January, February, and April at site 4. Minimum precipitation was recorded at sites 5 and 6, which was almost negligible. Whereas a moderate level of precipitation was recorded for sites 1, 2, and 3 throughout the wheat season except for the month of March. On the other hand, the maximum temperature was observed at sites 5 and 6, while the minimum temperature was recorded at site 4 (**Figure 5**).

### Correlation between growth parameters, soil physicochemical attributes, and meteorological factors

**Figure 6:**
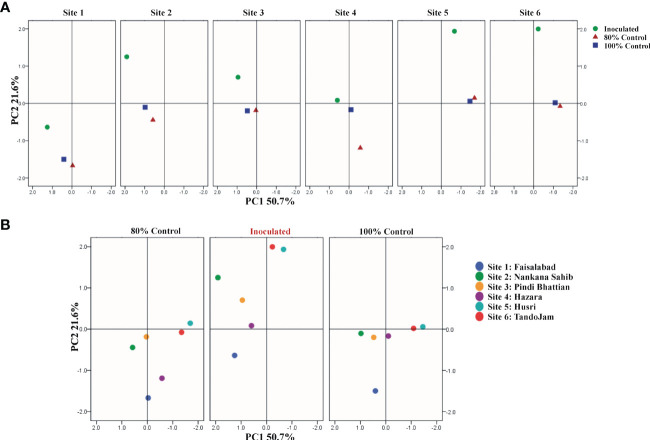
Principal component analysis (PCA) of wheat varieties inoculated with PSB and reduced application of DAP; treatment-wise **(A)** and location-wise analysis **(B)**. Treatments: inoculation of soil-specific consortia and uninoculated controls. Site 1: Faisalabad, Site 2: Nankana Sahib, Site 3: Pindi Bhattian, Site 4: Hazara, Site 5: Husri, and Site 6: Tando Jam.

Plant growth parameters were subjected to categorical principal component analysis (CAT-PCA). The PCA plot showed the correlation between the wheat yield parameters, with the two principal components (PCs) contributing up to 73% to the variance on the x-axis (PC1 = 51%) and y-axis (PC2 = 22%). Inoculated plants had a significant (positive) effect on grain yield, plant tillers, soil-available P, soil phosphatase activity, and seed P content. No parameter was found negatively affected by the PSB inoculation. The analysis demonstrated the treatment differences in all six soils. Among the six soils, the effect of treatments was pronounced at sites 2, 3, 5, and 6. PCA showed a pronounced effect of soil-specific consortium on plant growth parameters and soil parameters (**Figure 6**). Regression analysis confirmed a positive correlation between wheat yield parameters, i.e., seed P content, wheat grain yield, and soil-available P as a result of PSB consortium application in multilocational field trials (**Figure S2**).

**Figure 7:**
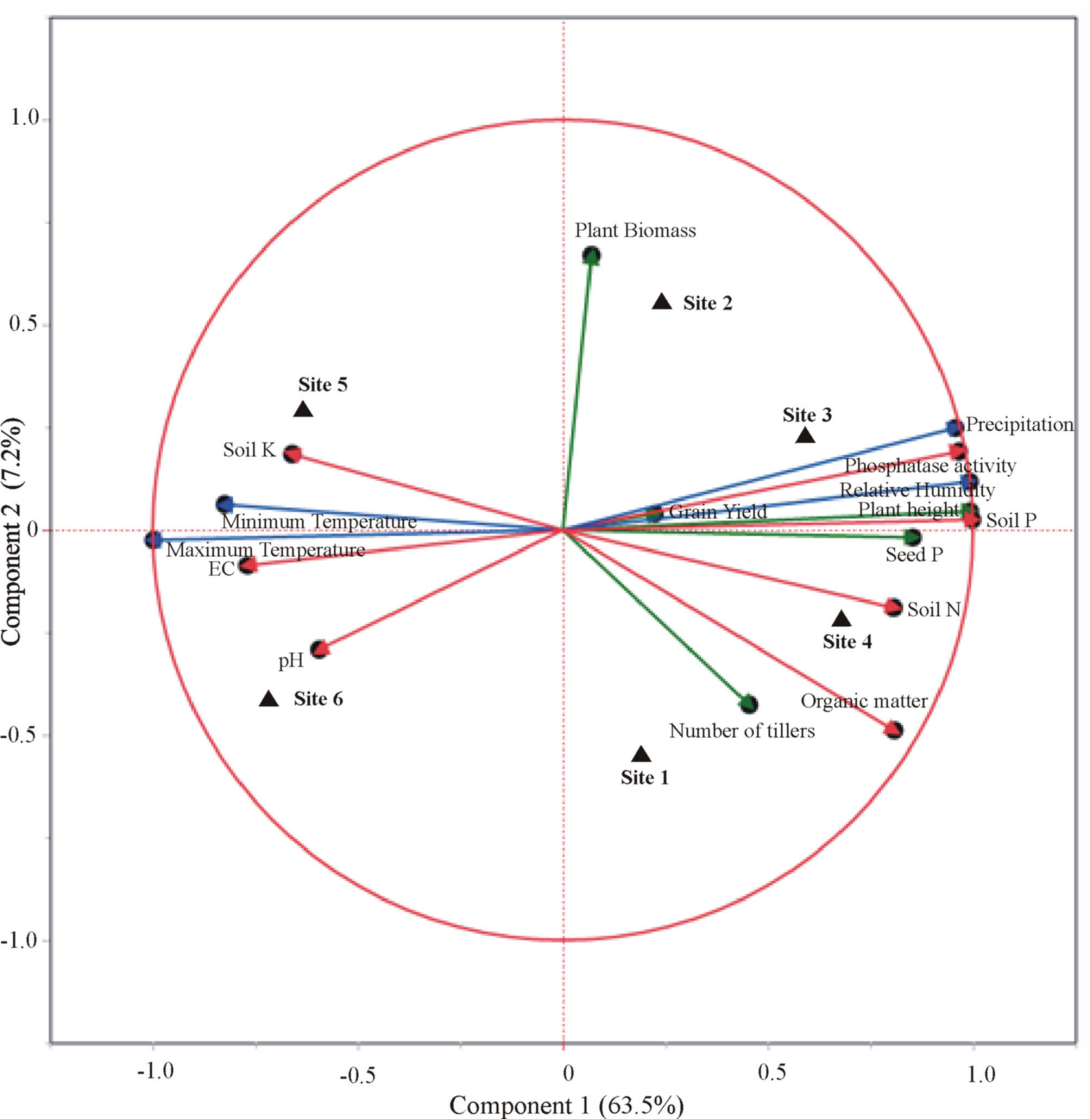
Categorical principal component analysis (CAT-PCA) of wheat yield parameters, seed P content, soil nutrient parameters, and climatic/meteorological conditions at the trial sites in response to inoculation of phosphate-solubilizing bacteria. Site 1: Faisalabad, Site 2: Nankana Sahib, Site 3: Pindi Bhattian, Site 4: Hazara, Site 5: Husri, and Site 6: Tando Jam.

Furthermore, CAT-PCA of wheat yield parameters, soil physicochemical analysis, and meteorological factors revealed that the success of each consortium was the result of varying factors associated with the meteorological conditions and soil nutritional status of that site (**Figure 7**). A total of 71% variation was explained by PCA, where 64% variance was accounted for by PC1 and 7% by PC2. A positive correlation of PSB-inoculated field-grown wheat to grain yield, soil P content, and precipitation was observed for sites 2 and 3 belonging to irrigated plains. While seed P content, soil organic matter, and number of tillers were found positively correlated with site 4 belonging to Northern dry mountains. However, the impact of inoculation at sites 5 and 6 belonging to the Indus delta was found considerably correlated to soil K content, EC, and temperature.

## Discussion

The burgeoning global biofertilizer market for agricultural use is driven by the pressure to surge sustainable crop production. The success of biofertilizers is primarily dependent upon the ability of inoculants to persist and perform effectively under natural environmental conditions (Lopes et al., 2021). Although an elite bacterial strain is essential for the efficacious development of inoculants, non-biological components are the foremost dynamics for the consistent performance of inoculum under field conditions (Mendoza-Suárez et al., 2021). Extensive field evaluation of inoculum has rarely been evaluated under a range of soils and environmental conditions and is urgently needed to foster successful implementation by farmers or growers. The potential impact of the environment on inoculation is usually neglected. Therefore, this study provides the first holistic report on the development of soil-specific PSB consortia and their application in respective agro-climatic conditions.

Three consortia were designed for their native soils and respective recommended wheat varieties by using the most efficient PSB having multiple plant growth-promoting traits such as indole acetic acid production, zinc solubilization, and siderophore production (Yahya et al., 2021). This is because native microorganisms are more adaptable and persist longer in native soils (Souza et al., 2015). A shelf-life study of three consortia up to 270 DPI indicated that these are the elite PSB strains and that FM had significantly maintained a higher bacterial load. This also suggests that the carrier material based on FM provides a more suitable microenvironment for inoculated PSB and has a longer shelf life. This is an essential property of carrier materials for maintaining microbial viability (Soumare et al., 2020).

Therefore, for designing the optimal inoculant formulation, a well-characterized FM-based carrier material was used in the study. Other contributing factors for the maintenance of microbial viability are the constitutional essential elements in FM, predominantly silicon (Si), iron (Fe), P, calcium (Ca), magnesium (Mg), carbon (C), and oxygen (O) (Yahya et al., 2022). Studies showed that a significant amount of Si, Fe, P, Ca, and Mg in FM made it a suitable product as a source of nutrients (Dotaniya et al., 2016).

To investigate the contribution of these PSB bioformulations to crop yield, a pot experiment was performed with Faisalabad 2008 variety of wheat grown under net house conditions. Significant increase (up to 1.4%) in grain yield, plant biomass (1-1.3%), seed P content (up to 4.32%), and soil phosphatase activity (up to 24%) and subsequent P availability in the soil (up to 6.25%) was observed in inoculated plants treated with reduced (20%) application of DAP.

The survivability of inoculated PSB in wheat rhizosphere was verified by viability and FISH, indicating that inoculated PSB were rhizosphere-competent phosphobacteria. Furthermore, the P-solubilizing ability of reisolated PSB was compared to their pure cultures, indicating the persistence of inoculated PSB. Morphologically similar reisolated colonies of SSR obtained from inoculated treatments were further validated by amplification of the *gcd* gene (MK883703) specific for *Ochrobactrum* strain SSR (Rasul et al., 2021). As SSR is one of the most potent strains for which strain-specific primers were available. Persistent colonization of PGPR in the rhizosphere indicates that bacteria can perform their functions (Lopes et al., 2021) and form associations with local microbial communities (Santoyo et al., 2021).

The P-solubilizing efficacy of the three PSB consortia was further evaluated under field conditions in their respective wheat-growing areas and recommended wheat varieties. The results showed an increase in grain yield (2%–14%) and seed P content (3%–5%) in inoculated treatments with a reduced application of DAP as compared to uninoculated controls. Previous studies indicated that P-solubilizing microorganisms showed the best effect with reduced application of DAP fertilizers (Rasul et al., 2019; Rosa et al., 2020; Yahya et al., 2022). Higher seed P content might be due to P translocation to seed because of PSB inoculation (Feng et al., 2021). Maximum grain yield (5,390 kg ha^-1^) was observed as a result of consortium-1 inoculation at site 2 followed by site 3 (5,240 kg ha^-1^) and site 1 (4,806 kg ha^-1^). In the case of consortium-2, a grain yield of 5,174 kg ha^-1^ was observed at site 4 with a 20% reduced application of DAP. An increase in grain yield (5,324 kg ha^-1^) was observed as a result of consortium-3 inoculation at site 6 followed by site 5 (4,806 kg ha^-1^).

CAT-PCA of wheat yield parameters, soil physicochemical analysis, and meteorological factors revealed a positive correlation of PSB-inoculated field-grown wheat to grain yield, soil P content, and precipitation at irrigated plains, while seed P content, soil organic matter, and number of tillers were found positively correlated to sites belonging to the northern dry mountains. However, the impact of inoculation at sites belonging to the Indus delta was found to correlate with soil K content, EC, and temperature. The higher grain yield at site 4 may be due to higher soil organic matter and N contents that favor the persistence of inoculated PSB in dry mountainous soils. For instance, the organic matter of site 2 was higher (0.6%) as compared to that of site 3 and site 1 belonging to the irrigated plains. Similarly, the organic matter of site 6 was higher (0.56%) as compared to site 5 belonging to the Indus delta. This is due to the soils having a high organic matter that have higher microbial dynamics and thus eventually need lesser requirements for chemical fertilizers (Backer et al., 2018). The increase in soil organic matter is the key factor in maintaining soil fertility and plant nutrient uptake (Gerke, 2022). This can sustain agricultural productivity by restricting the use of chemical fertilizers (Allam et al., 2022). Therefore, it could be more important to amend the soil with organic matter instead of seed pelleting to augment the soil with appropriate soil fertility. Other than organic matter, soil pH, carbon content, and water availability are the important determinants for successful inoculum survival in soil under field conditions (Hartmann et al., 2015; Mahoney et al., 2017; Cao et al., 2021; Gao et al., 2021). It also depends on the soil type and the growing season (Bolyen et al., 2019; Khandare et al., 2020; Yan et al., 2020). Hence, it is essential to take into account all of these factors so that bacteria can colonize efficiently in native environmental conditions.

Secondly, the other factors that can contribute to wheat production are the climatic conditions, i.e., temperature (minimum and maximum), rainfall, relative humidity, and sunshine. These climate changes directly affect the productivity and stability of the agriculture sector (Lobo et al., 2019). Studies have shown that the most influential climatic factors in wheat production in Pakistan are relative humidity, maximum temperature, and rainfall. Maximum temperature negatively influenced the wheat yield (Ghani et al., 2021). In this study, a similar trend was observed, for example, the minimum yield was observed for sites 5 and 6, which have maximum average temperature throughout the wheat season, whereas the minimum temperature is reported to have a significant positive impact on wheat yield. Likewise, the minimum temperature was observed at site 4 with concomitant enhanced wheat yield. On the other hand, precipitation can influence the effectiveness of biofertilizers, which usually depends on soil properties. Biofertilizers are more effective in arid climates than in snowy climates (Jennifer et al., 2018). In the present study, a positive correlation of field-grown PSB-inoculated wheat with grain yield, soil P content, and precipitation was observed for sites 2 and 3, which belong to the semiarid zone. However, bacterial communities can be distinct for each site or ecosystem along the precipitation gradient (Bachar et al., 2010).

Soil microbial activity largely depends upon the temperature and soil moisture level in rain-fed agriculture (Cookson et al., 2002). The practical implication of moisture and temperature requirements is needed optimally around the establishment of crops in Mediterranean climates (Gupta et al., 2011). Studies indicated that the successful use of inoculants can only be possible for arid environments when they are applied in a timely manner (Rubin et al., 2017; Chandran et al., 2021), since rapid wetting and drying cycles can be detrimental to the survival of the inoculum (Vriezen et al., 2007). Therefore, the inoculum must be applied to soil when the moisture content is adequate for seed germination and colony propagation.

Knowing the soil’s nutritional status guides the sensible use of the inoculum. Subsequently, it is important to identify soil deficiencies in concert with the application of the inoculum. The interaction between soil C and N should also be considered, since inoculants capable of building soil organic C and improving soil structure only have this potential if soil-available N is adequate (Callaghan et al., 2022). Soil properties that can disrupt the microbial community and strategic cultivation could provide an opportunity to balance the soil conditions in favor of inoculated microbe. Hence, this study is of significant worth, and for the first time, it reports the development and application of soil-specific biofertilizers for agroecological zones of wheat. Meanwhile, it integrates soil nutritional status and agro-climatic conditions simultaneously which are found to be the key factors for consistent performance of augmented PSB. The potential *gcd* gene containing phosphobacteria used in the study was found promising for P biofortification; therefore, these might be used in the future for the development of potential biofertilizers to foster sustainable wheat production in diverse agro-climatic zones.

## Conclusion

Despite the significance of the growing biofertilizer market, microbial inoculants still failed to deliver on their potential except for a few products. To the best of our knowledge, this study provides innovative insights into the imminent significance of soil-specific biofertilizers for sustainable wheat production by integrating soil nutritional status and meteorological conditions at the site of application. These consortia were found promising for P biofortification; hence, these will be used for the development of potential biofertilizers. However, the persistence and efficacy of inoculated microbe are the key components to harnessing their potential. Therefore, targeted application of biofertilizers in native soils will provide a sound basis for the efficacious inoculants.

Furthermore, new approaches like metabarcoding should be opted for the selection of a potential native PGPR consortium that can survive and establish in complex microbial communities. Research priorities are needed to allow greater exploitation of microbiomes in sustainable agriculture including core microbiomes and metagenomes of target crops. Nevertheless, metabarcoding is a powerful tool to estimate soil microbial biodiversity. There is a dire need to integrate other crucial environmental factors to obtain a full picture of biodiversity attributes that can influence the functioning of ecosystems. This could lead to developing potential soil-specific consortia with concomitant adaptability under native agro-climatic conditions and soil nutritional status.

## Data availability statement

The original contributions presented in the study are included in the article/**Supplementary Material**. Further inquiries can be directed to the corresponding authors.

## Author contributions

MY analyzed the data and wrote the manuscript. SY and TW performed data analysis and review the manuscript. SZ performed SEM analysis. AD helped in making the map. MU, LR, AA and MA helped to conduct multilocation trials. MY executed statistical analysis. SY conceived and supervised the whole study and edited the manuscript. All authors contributed to the article and approved the submitted version.
