# Supplementary figures and images for: Integrated analysis of potential microbial consortia, soil nutritional status, and agro-climatic datasets to modulate P nutrient uptake and yield effectiveness of wheat under climate change resilience
Source: Front Plant Sci. 2023 Jan 12;13:1074383. doi: 10.3389/fpls.2022.1074383 (PMC9878846; doi:10.3389/fpls.2022.1074383)

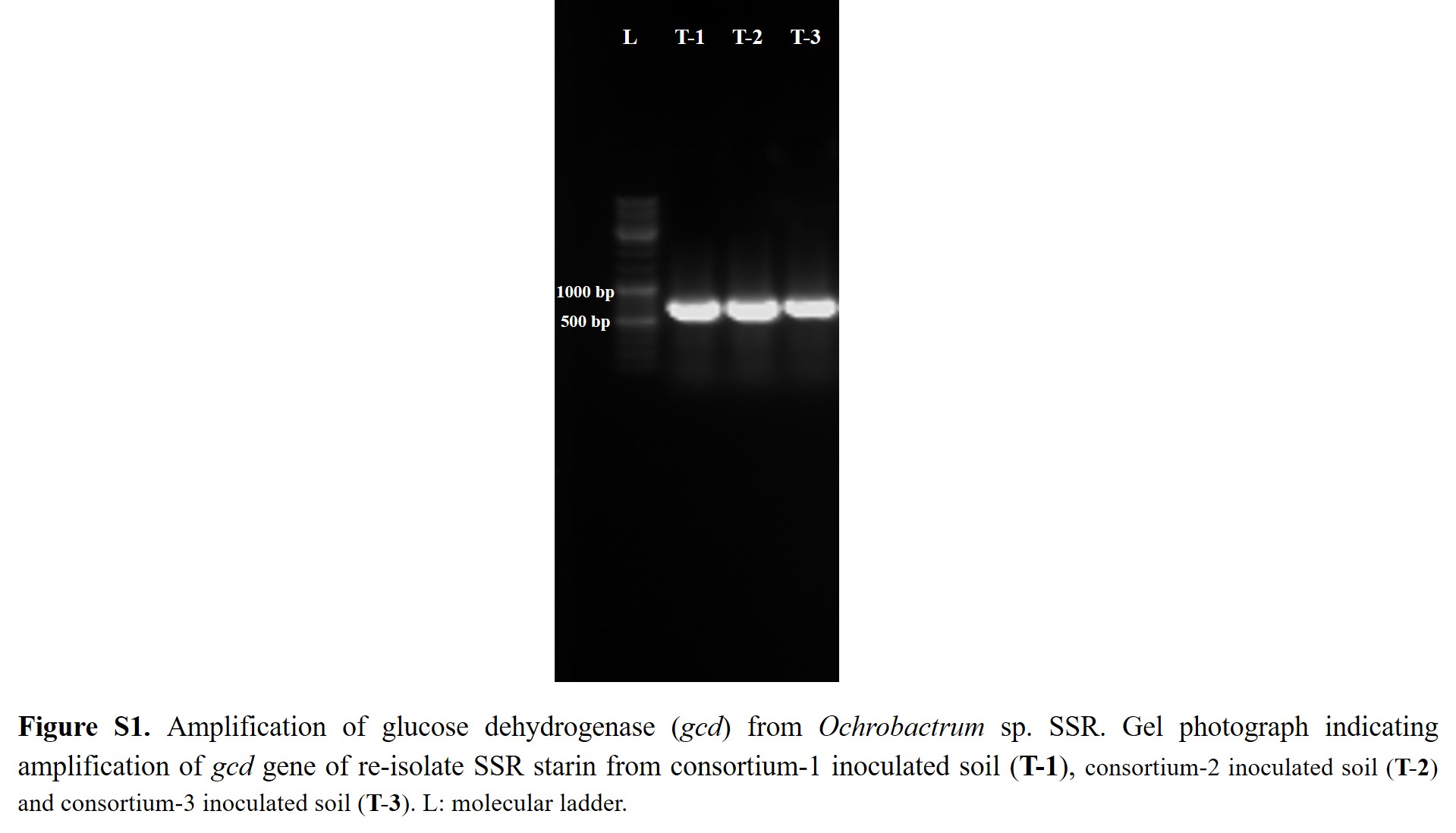

Supplement: Supplementary file 1 [file Image_1.jpg]

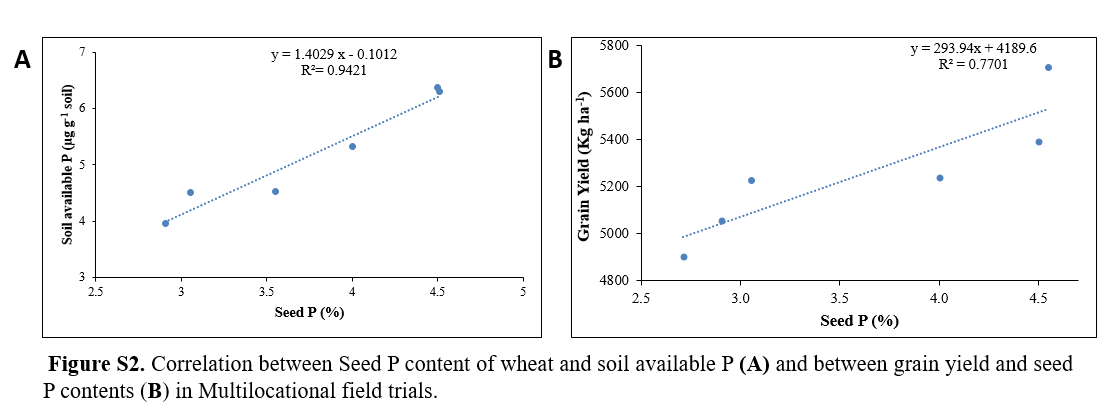

Supplement: Supplementary file 2 [file Image_2.png]
