# Supplementary material for: Integrated analysis of potential microbial consortia, soil nutritional status, and agro-climatic datasets to modulate P nutrient uptake and yield effectiveness of wheat under climate change resilience
Source: Front Plant Sci. 2023 Jan 12;13:1074383. doi: 10.3389/fpls.2022.1074383 (PMC9878846; doi:10.3389/fpls.2022.1074383)
Supplement: Supplementary file 3 [file DataSheet_1.pdf]

Table S1. Viable count of Filter mud based formulation with soil-specific consortia

Days Post Inoculation	Viable Count (CFU g^{-1})		
	Consortium-1	Consortium -2	Consortium -3
**15**	4.32×10^{8}	3.39×10^{8}	5.05×10^{7}
**30**	4.72×10^{7}	3.27×10^{7}	3.20×10^{6}
**60**	3.70×10^{7}	2.49×10^{7}	3.13×10^{6}
**90**	3.69×10^{7}	2.35×10^{7}	2.06×10^{6}
**180**	3.65×10^{7}	2.24×10^{7}	5.35×10^{5}
**270**	2.51×10^{7}	2.10×10^{7}	5.26×10^{5}

All values are an average of six biological replicates.

Table S2. Effect of Phosphate solubilizing bacteria on wheat growth, plant P and soil nutrients in a pot experiment under net house conditions

Treatments	35 DAS							120 DAS						
	**Plant weight (g plant^{-1})**	**Shoot Length (cm)**	**Root Length (cm)**	**Plant P content (%)**	**Soil Available P (µg g^{-1} soil)**	**Phosphatase Activity (µmole g^{-1} soil hr^{-1})**	**Viable (CFU g^{-1}soil)**	**No. of tillers (tillers plant^{-1})**	**Shoot Length (cm)**	**Plant Biomass (g plant^{-1})**	**Grain Yield (g plant^{-1})**	**Seed P content (%)**	**Soil Available P (µg g^{-1} soil)**	**Phosphatase Activity (µmole g^{-1} soil hr^{-1})**
**Control 100%**	0.80±0.04 C	20.33±1.10 C	08.53±0.42 B	2.07±0.15 D	3.41±0.16 C	6.93±0.34 C	3.88×10^{7}	8.67±0.45 C	76.12±3.80 A	7.86±0.21 C	5.50±0.30 A	3.44±0.12 B	2.46±0.11 C	15.59±0.88 B
**Control 80%**	0.51±0.03 D	18.73±0.93 C	06.77±0.33 C	0.97±0.04 E	3.27±0.17 C	6.53±0.32 C	2.26×10^{7}	5.00±0.25 D	71.81±3.59 A	4.31±0.39 D	2.43±0.13 B	2.07±0.14 C	2.27±0.12 C	12.90±0.65 C
**Cons-1 (80%)**	1.87±0.09 A	37.25±1.86 A	12.00±0.61 A	4.62±0.25 A	6.40±0.38 A	12.13±0.65 A	9.81×10^{7}	10.83±0.54 A	77.22±3.86 A	10.21±0.50 A	5.95±0.30 A	4.32±0.25 A	6.25±0.31 A	24.43±1.24 A
**Cons-2 (80%)**	1.81±0.09 A	34.67±1.73 B	11.33±0.58 A	3.73±0.19 C	6.08±0.24 AB	11.05±0.55 B	9.39×10^{7}	09.67±0.48 B	77.32±3.85 A	8.83±0.44 B	5.83±0.29 A	3.63±0.18 B	5.96±0.30 AB	23.00±1.15 A
**Cons-3 (80%)**	1.63±0.08 B	33.00±1.65 B	11.00±0.50 A	4.08±0.20 B	5.65±0.18 B	10.60±0.53 B	9.13×10^{7}	09.00±0.46 BC	76.51±3.84 A	8.28±0.41 BC	5.77±0.29 A	3.61±0.18 B	5.76±0.29 B	22.97±1.15 A

[1,2] Effect of bacterial inoculation on wheat growth parameters, P uptake and soil nutrients at 35 days after sowing (DAS) and at 120 DAS in a pot e×periment at Faisalabad grown wheat during winter season 2018.Control 80% and Control 100% represents non-inoculated controls supplemented with 80% of the recommended dose of P fertilizer and full recommended dose of P fertilizer, respectively. PSB (1×10^{9} CFU mL^{-1}) were seed-inoculated before sowing. Inoculated pots were supplemented with 80% of recommended dose of DAP fertilizer. Means are the average of six replicates arranged in CRD. Means followed by the same letter differ non-significantly at $p = 0.05$ according to LSD. ± represents the standard deviations (SD). Plants were grown in earthen pots (Diameter: 12 inch, Height: 14 inch) containing 12 kg soil.
